# Resolvin E1-ChemR23 Axis Regulates the Hepatic Metabolic and Inflammatory Transcriptional Landscape in Obesity at the Whole Genome and Exon Level

**DOI:** 10.3389/fnut.2021.799492

**Published:** 2021-12-24

**Authors:** Abrar E. Al-Shaer, Anandita Pal, Saame Raza Shaikh

**Affiliations:** Department of Nutrition, Gillings School of Global Public Health and School of Medicine, The University of North Carolina at Chapel Hill, Chapel Hill, NC, United States

**Keywords:** resolvin E1, ChemR23, ERV1, exon level analysis, hepatic transcriptome, eicosapentaenoic acid, obesity

## Abstract

Resolvin E1 (RvE1) is an immunoresolvent that is synthesized from eicosapentaenoic acid and can bind the receptor ERV1/ChemR23. We previously showed activation of the RvE1-ChemR23 axis improves hyperglycemia and hyperinsulinemia of obese mice; however, it remains unclear how RvE1 controls glucose homeostasis. Here we investigated hepatic metabolic and inflammatory transcriptional targets of the RvE1-ChemR23 axis using lean and obese wild type (WT) and ChemR23 knockout (KO) mice. We conducted an in-depth transcriptional study by preforming whole gene-level and exon-level analyses, which provide insight into alternative splicing variants and miRNA regulation. Compared to controls, WT and KO obese mice in the absence of RvE1 displayed similar gene-level profiles, which entailed dysregulated pathways related to glucose homeostasis. Notably, obese WT mice relative to lean controls showed a robust decrease in pathways related to the biosynthesis of unsaturated fatty acids. At the exon-level, obese ChemR23 KOs compared to obese WT mice displayed changes in pathways related to hepatic lipid transport, cholesterol metabolism, and immunological functions such as complement cascades and platelet activation. Importantly, upon RvE1 administration to WT obese mice, we discovered upregulated genes in pathways relating to insulin sensitivity and downregulated genes related to regulators of TGF-β signaling. This transcriptional profile was generally not recapitulated with obese ChemR23 KO mice administered RvE1. Collectively, gene and exon-level analyses suggest RvE1 controls the hepatic transcriptional profile related to glucose homeostasis, insulin sensitivity, and inflammation in a manner that is largely dependent on ChemR23. These studies will drive future mechanistic experiments on the RvE1-ChemR23 axis.

## Introduction

Specialized pro-resolving mediators (SPMs) are highly potent oxylipins that are enzymatically synthesized from polyunsaturated fatty acid (PUFAs) (1–3). SPMs of the resolvin, protectin, and maresin family are predominately generated from the long chain n-3 PUFAs eicosapentaenoic (EPA) and docosahexaenoic (DHA) acids (2). SPMs known as lipoxins can also be produced from arachidonic acid, an n-6 PUFA (4, 5). Functionally, SPMs have a critical role in driving the resolution of inflammation and returning damaged tissues to homeostasis across a range of diseases (2, 6). Notably, increasing the concentration of SPMs, either through the diet with PUFA consumption or upon direct SPM administration, improves several complications associated with diet-induced obesity (2, 7–10). These complications include atherosclerosis, insulin resistance, wound healing, and even the response to viral infection (7, 10–13).

Several research groups have reported the importance of the resolvin E1 (RvE1)-ERV1/ChemR23 axis for improving metabolic outcomes related to obesity (10, 12, 14, 15). ERV1/ChemR23 is of interest as it is G-protein coupled receptor that binds RvE1 in addition to the adipokine chemerin (16, 17). To exemplify the importance of this pathway, targeted deletion of ERV1/ChemR23 in mouse models of hyperlipidemia drove pro-atherogenic signaling, which was reversed upon activation of this pathway with either dietary EPA intervention or treatment with RvE1 (12). Gain-of-function studies also underscore the importance of this pathway; for instance, overexpression of ERV1/ChemR23 in myeloid cells prevented hyperglycemia and hepatic steatosis (15). Our laboratory previously reported that obesity drove a tissue-specific deficiency in 18-hydroxyeicosapentaenoic acid, which is the precursor for RvE1 (10). Importantly, RvE1 administration to obese mice improved hyperinsulinemia and hyperglycemia in an ERV1/ChemR23-dependent manner (10).

There are several cellular mechanisms by which SPMs such as RvE1 exert their biological activity. For instance, RvE1, similar to other resolvins, triggers phenotypic switching of macrophage polarization toward a pro-resolution phenotype (18). Furthermore, RvE1 improves the phagocytic capacity of neutrophils in models of type 2 diabetes (14). However, a major gap in knowledge of our understanding of SPMs including RvE1 is how do these potent metabolites control the genome. This is an important question as there is increasing appreciation that diseases such obesity are highly heterogenous and therapeutic approaches are likely to dependent on the interactions between a given intervention and the host genome (19). Therefore, in this study, we used obese C57BL/6J and ERV1/ChemR23 knockout (KO) mice to study the role of ChemR23 in controlling the liver transcriptome. We focused on the liver given its critical function in maintaining glucose homeostasis. First, we studied the impact of the high fat diet and the loss of ChemR23 itself on the hepatic transcriptional landscape relative to controls. Next, we investigated the role of RvE1 on the liver transcriptome in obese WT and KO mice. The approach relied on gene and exon-level RNAseq analyses, which provided a deeper analysis at the level of gene splicing variants and miRNA regulation. These studies provide the first evidence of how the RvE1-ChemR23 axis controls the hepatic genome in the context of diet-induced obesity.

## Materials and Methods

### Animal Models

All murine experiments followed IACUC guidelines established by The University of North Carolina at Chapel Hill. Euthanasia was performed via isoflurane inhalation followed by cervical dislocation. The ChemR23 knockout (KO) mice were generated by the CRISPR/Cas9-mediated genome editing on a C57BL/6J background as described in our previous work (10). All studies relied on wild type (WT) littermate controls. Male ChemR23 KO and WT mice were fed a lean control (10% kcal from lard, Envigo TD.160407) or high-fat (HF) (60% kcal from lard, Envigo TD.06414, Indianapolis, IN) diet starting at 6 weeks of age for 15 consecutive weeks.

### RvE1 Treatment & Tissue Harvesting

After 15 weeks of intervention with a high fat diet, mice were intraperitoneally administered either 300ng of RvE1 or a vehicle control for four consecutive days as previously described (10). All tissues were harvested on day 5 after the four days of administration were completed. All mice were fasting for at least four hours upon tissue harvest, and livers were dissected, and flash frozen in liquid nitrogen and then stored at −80°C for RNA sequencing. A total of seven mice per group (WT lean, WT obese, KO lean, KO obese, WT obese+RvE1, KO obese+RvE1) were used for RNA-sequencing. Mice that were not injected with RvE1 received PBS+ethanol vehicle control injections. The ETOH concentration in the vehicle controls matched the RvE1 solvent solution.

### Liver RNA Sequencing

All RNA analytes were assayed for RNA integrity, concentration, and fragment size. Samples for total RNA-Seq were quantified on a TapeStation system (Agilent, Inc. Santa Clara, CA, USA). RNA Integrity score (RIN) averaged at 7.4. Samples with RINs > 7.0 were considered high quality. Total RNA-Seq library construction was performed from the RNA samples using the TruSeq Stranded RNA Sample Preparation Kit and bar-coded with individual tags following the manufacturer's instructions (Illumina, Inc. San Diego, CA). Libraries were prepared on an Agilent Bravo Automated Liquid Handling System. Quality control was performed at every step and the libraries were quantified using a TapeStation system. Indexed libraries were prepared and run on the NovaSeq 6000 to generate an average of 35 million reads per sample. The raw Illumina sequence data were demultiplexed and converted to FASTSQ files, and adapter and low-quality sequences were quantified.

### Gene-Level Analyses

Each Fastq file's quality was checked using FastQC. All Fastq sequence files were aligned to the *Mus musculus* UCSC MM10 reference genome using Bowtie2 (20). The outputted SAM files were converted to BAM files for downstream analysis using the subread featureCounts package (21). We included a stringency parameter (-B) to ensure mapping of both paired ends when assigning counts to a specific gene. DESeq2 was used for normalization and statistical analysis of the gene counts output from the featureCounts program. DESeq2 is a conservative software that utilizes a negative binomial distribution with a generalized linear model to adjust for false positive significance values that result from running statistical tests on tens of thousands of genes (22). The DESeq2 analysis generated Benjamini-Hochberg (BH) adjusted p-values, log2 fold changes, and normalized counts for each gene. All further statistical analyses only utilized BH adjusted *p*-values below 0.1, or a false discovery rate (FDR) of 10%. Pathway level analysis was performed via the DAVID Functional Annotation Tool v6.8 from the exon generated data.

### Exon-Level Analyses

Similar to the gene-level analysis, all Fastq sequence files were aligned to the *Mus musculus* UCSC MM10 reference genome using Bowtie2 (20). The outputted SAM files were converted to BAM files for downstream analysis using the subread featureCounts package (21). We included the stringency parameter (-B) to ensure mapping of both paired ends when assigning counts to a specific exon and we used the -f parameter to specify feature (exon) level counts and not metafeature (gene) level counts. For normalization and statistical analysis, we used the DEXSeq package, a sister package to DESeq2 that also utilizes a conservative approach to adjust for false positives in the statistical output (23). The DEXSeq analysis generated Benjamini-Hochberg (BH) adjusted *p*-values, log2 fold changes, and normalized counts for each exon. All further statistical analyses only utilized BH adjusted *p*-values below 0.1, or a false discovery rate (FDR) of 10%. Exon usage splicing plots were generated by DEXSeq and represent significant changes in the expression of individual exons that are not due to the overall up- or down-regulation of the gene (23). Gene ontology molecular function analysis was performed via the DAVID Functional Annotation Tool v6.8 from the exon generated data.

### miRNA Target Prediction Analysis

TargetScanMouse 7.2 was used to predict possible gene-pathway targets for each of the identified miRNAs from the exon-level analysis. The TargetScan tool assigns a “cumulative weighted context score++” which ranks the list of predicted miRNA gene targets from most likely to least likely to be a true positive target (24). The more negative the score the more likely the predicted miRNA gene target is a true positive. We filtered all the TargetScan results to only include predicted targets with a cumulative weighted context score++ ≤−0.4 (25). The filtered list of miRNA gene targets was then inputted in the DAVID Functional Annotation Tool v6.8 to cluster the gene targets into their respective KEGG pathway or gene ontology annotations. All generated graphs were sorted by *p*-value.

## Results

### Diet-Induced Obesity Dysregulates Metabolic Gene Transcripts in Wildtype and ChemR23 Knockout Mice

**Figure 1:**
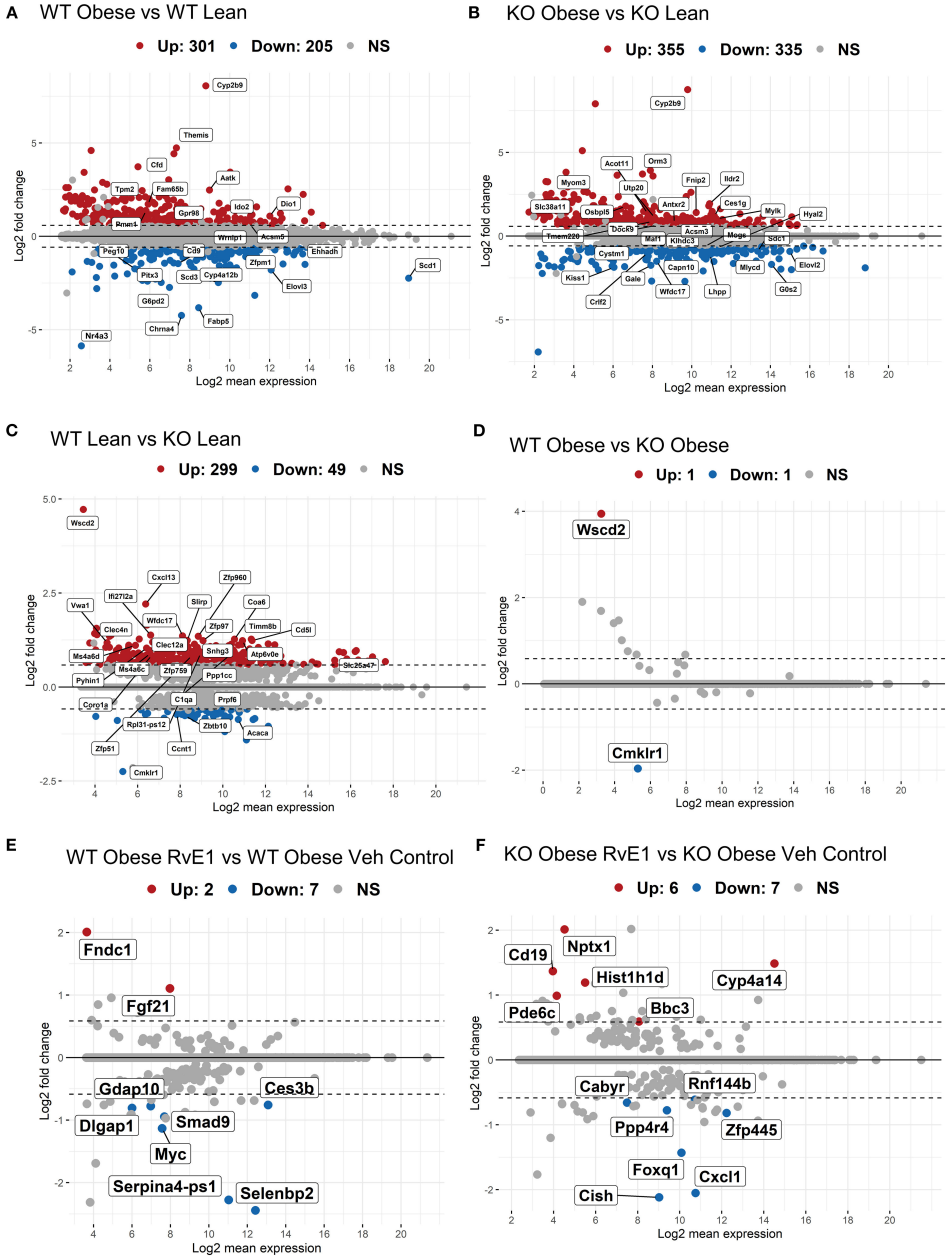
Obese WT and ChemR23 KOs in the absence and presence of RvE1 display differential expression of liver metabolic and immunological transcripts. MA plots showing genes with a BH-adjusted *p*-value below 0.1 and Log2 fold change (FC) above 1.5 plotted by Log2FC in the y-axis and Log2 mean expression in the x-axis. Upregulated genes are colored in red and downregulated genes are colored in blue. Non-significant genes are colored in gray and denoted as NS. **(A)** MA plot of differentially expressed genes between WT obese and WT lean mice. **(B)** MA plot of differentially expressed genes between ChemR23 KO obese and ChemR23 KO lean mice. **(C)** MA plot of differentially expressed genes between WT lean and ChemR23 KO lean mice. **(D)** MA plot of differentially expressed genes between WT obese and ChemR23 KO obese mice. **(E)** MA plot of differentially expressed genes between WT obese+RvE1 treated mice and WT obese+vehicle control treated mice. **(F)** MA plot of differentially expressed genes between ChemR23 KO obese+RvE1 treated mice and ChemR23 KO obese+vehicle control treated mice. Data are from 7 mice per group.

We first analyzed the hepatic transcriptome of wildtype (WT) obese vs WT lean mice. WT mice fed a high fat diet compared with WT lean controls had 301 upregulated genes and 205 downregulated genes with a BH-adjusted p-value below 0.1 and Log2 fold change (FC) above 1.5 (Figure 1A). The most upregulated genes in WT obese mice were cytochrome P450 family 2 subfamily C member 9 (Cyp2b9), complement factor D (Cfd), apoptosis-associated tyrosine kinase (Aatk), tropomyosin 2 (Tpm2). Collectively, Cyp2b9 (Log2FC = 8.05), Cfd (Log2FC = 4.42), Aatk (Log2FC = 2.47), and Tpm2 (Log2FC = 2.11) are reported to be upregulated with a high-fat diet and are implicated as markers of diet-induced hepatic steatosis, promotion of lipid accumulation, high-fat diet induced apoptosis, and atherosclerosis (26–29). On the other hand, the most downregulated gene transcripts included nuclear receptor subfamily 4 group A member 3 (Nr4a3), stearoyl-coA desaturase (Scd1), cholinergic receptor nicotinic alpha 4 subunit (Chrna4), and fatty acid binding protein 5 (Fabp5). Loss or downregulation of Nr4a3 (Log2FC = −5.87), Scd1 (Log2FC = −2.24), Chrna4 (Log2FC = −4.23), and Fabp5 (Log2FC = −3.82) increase glucose intolerance, insulin resistance, diet-induced fatty liver disease, and cholinergic anti-inflammatory pathways (30–32). Overall, these results confirm, as expected, that high fat diet induced obesity dysregulates genes related to hepatic metabolic regulation.

We next analyzed the transcriptional profile of obese ChemR23 KOs compared to lean ChemR23 KO mice. ChemR23 KO mice fed a high fat diet compared with ChemR23 KO lean controls had 355 upregulated genes and 335 downregulated genes with a BH-adjusted *p*-value below 0.1 and Log2 FC above 1.5 (Figure 1B). The most upregulated gene with a high-fat diet was Cyp2b9 (Log2FC = 8.74). Other significantly upregulated genes were orosomucoid 3 (Orm3, Log2FC = 3.70) and Acyl-CoA Thioesterase 11 (Acot11, Log2FC = 1.36) which are involved in energy expenditure, accumulation of fat mass, and insulin resistance (33, 34). Downregulated genes such as kisspeptin (Kiss1, Log2FC = −1.87), UDP-galactose-4-epimerase (Gale, Log2FC = −1.74), and G0/G1 switch gene 2 (G0s2, Log2FC = −1.69) have been linked to hepatic-regulated glucose homeostasis, insulin resistance, and adiposity (35–37). Thus, these results show obese ChemR23 KO mice have some dysregulated genes related to fat mass and insulin/glucose regulation compared to the controls.

### Differential Expression of Genes Between Lean WT and Lean ChemR23 KO Mice but Not Obese Mice

WT lean mice compared to ChemR23 KO lean mice had 299 upregulated genes and 49 downregulated genes with a BH-adjusted *p*-value below 0.1 and Log2 FC above 1.5 (Figure 1C). As expected, the most downregulated gene in the KO mice is Cmklr1 (ChemR23, Log2FC = −2.25), and the most upregulated gene is Wscd2 (Log2FC = 4.72), a gene enhancer/promoter for ChemR23. Other significantly upregulated genes in the KO mice comprised primarily of activated immunological and chemotaxis markers such as C-X-C motif chemokine ligand 13 (Cxcl13, Log2FC = 2.2), interferon alpha-inducible protein 27 like 2A (Ifi27l2a, Log2FC = 1.38), and the WAP four-disulfide core domain gene which encodes the activated macrophage/microglia WAP domain protein (Wfdc17, Log2FC = 1.36) (38–40). There were few downregulated transcripts in the KO lean mice which were notably involved in adipocyte differentiation, such as acetyl-CoA carboxylase α (Acaca, Log2FC = −0.86) and cyclin T1 (Ccnt1, Log2FC = −0.73) (41, 42). In contrast, obese KO mice had no change in gene expression with a BH-adjusted *p*-value below 0.1 and Log2 FC above 1.5 that were significantly different from the obese WT mice except for the expected ChemR23 and Wscd2 genes (Figure 1D). In summary, these results reveal lean ChemR23 KO mice display a larger differential expression at the gene level than obese mice relative to controls.

### RvE1 Treatment Drives Differential Expression of Glucose Homeostatic Genes in a Manner That Is Mostly ChemR23-Dependent

The next set of analyses focused on the impact of RvE1 on the hepatic transcriptome. WT obese mice administered RvE1 compared to WT obese mice given vehicle control had 2 upregulated genes and 7 downregulated genes with a BH-adjusted *p*-value below 0.1 and Log2 FC above 1.5 (Figure 1E). Significantly downregulated genes after RvE1 treatment such as MYC proto-oncogene (Myc, Log2FC = −1.13) and SMAD family member 9 (Smad9, Log2FC = −0.94) are key regulators in TGF-β signaling (43–45). In contrast, fibroblast growth factor 21 (Fgf21, Log2FC = 1.10) and fibronectin type III domain containing 1 (Fndc1, Log2FC = 2.0), associated with insulin sensitivity and hepatic steatosis, were significantly upregulated after RvE1 treatment (46–49).

ChemR23 KO obese mice administered RvE1 compared to ChemR23 KO obese mice given vehicle controls had 6 upregulated genes and 7 downregulated genes with a BH-adjusted *p*-value below 0.1 and Log2 FC above 1.5 (Figure 1F). In the ChemR23 KO mice, significantly downregulated genes after RvE1 treatment such as forkhead box q1 (Foxq1, Log2FC = −1.43), cytokine inducible SH2 containing protein (Cish, Log2FC = −2.12), and C-X-C motif chemokine ligand 1 (Cxcl, Log2FC = −2.05) affect both hepatic glucose homeostasis and immune cell trafficking/activation (50–54). Significantly upregulated genes after RvE1 treatment such as differentiation antigen CD19 (Cd19, Log2FC = 1.37), neuronal pentraxin 1 (Nptx1, Log2FC = 2.01), phosphodiesterase 6C (Pde6c, Log2FC = 1.0), and BCL2 binding component 3 (Bbc3, Log2FC = 0.59) are associated with multiple pathways spanning hyperglycemia, p53 signaling, and apoptosis (46, 55, 56).

We also examined whether LBT4R, the BLT1 receptor, or other related genes (ALOX5, ALOX5AP, LTA4H) were changed upon RvE1 administration in obese WT or ChemR23 KO mice and found no significant differences (data not shown). Overall, these studies showed that RvE1 significantly alters expression of genes related to glucose homeostasis in a manner that was distinct between obese WT and ChemR23 KO mice.

### Diet and Genotype Effects on Differentially Expressed KEGG Pathway Clusters

**Figure 2:**
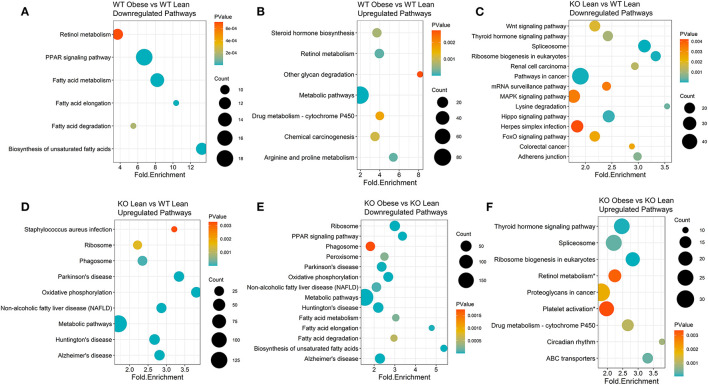
KEGG pathway clusters capture diet and genotype effects of differentially expressed genes transcripts of obese WT and ChemR23 KO mice. KEGG pathway clusters are listed for each comparison where the sizes of the dots on the plot indicate the number of genes in the KEGG pathway cluster, the p-value is denoted in a colored blue-orange scale, and each pathway is plotted by its fold enrichment value. **(A)** Downregulated KEGG pathways between WT obese and WT lean mice. **(B)** Upregulated KEGG pathways between WT obese and WT lean mice. **(C)** Downregulated KEGG pathways between ChemR23 KO lean and WT lean mice. **(D)** Upregulated KEGG pathways between ChemR23 KO lean and WT lean mice. **(E)** Downregulated KEGG pathways between ChemR23 KO obese and KO lean mice. **(F)** Upregulated KEGG pathways between ChemR23 KO obese and ChemR23 KO lean mice. Data are from 7 mice per group.

To generate KEGG pathway clusters, we next examined all upregulated and downregulated genes with a BH-adjusted *p-*value < 0.1, regardless of fold change. WT obese mice, compared to WT lean controls, had the most significant downregulation of biosynthesis of unsaturated fatty acids with a fold enrichment of 13.3 (Figure 2A) and the most significant upregulation of glycan degradation with a fold enrichment of 8.15 (Figure 2B). Lean KO mice exhibited significant downregulation of lysine degradation (fold enrichment = 3.54), ribosomal biogenesis (fold enrichment = 3.32), and spliceosome pathways (fold enrichment = 3.11) compared to lean WT mice (Figure 2C). In addition, lean KO mice had significant upregulated pathways in oxidative phosphorylation (fold enrichment = 3.81) and non-alcoholic fatty liver disease (NAFLD) (fold enrichment = 2.86) compared to lean WT mice (Figure 2D). As for the obese KO mice, we found significant downregulation of biosynthesis of unsaturated fatty acids (fold enrichment = 5.37) and fatty acid elongation (fold enrichment = 4.78) compared to lean KO mice (Figure 2E). The obese KO mice also had significant upregulation of circadian rhythm (fold enrichment = 3.78) and ABC transporter (fold enrichment = 3.31) pathways (Figure 2F). There were not enough significantly differentially expressed genes between the RvE1 treated groups to conduct a KEGG pathway clustering analysis. Nevertheless, these results revealed notable differences between lean and obese WT and KO mice.

### Exon Level Analyses Reveal Higher Resolution of the RvE1-ChemR23 Transcriptional Landscape

**Figure 3:**
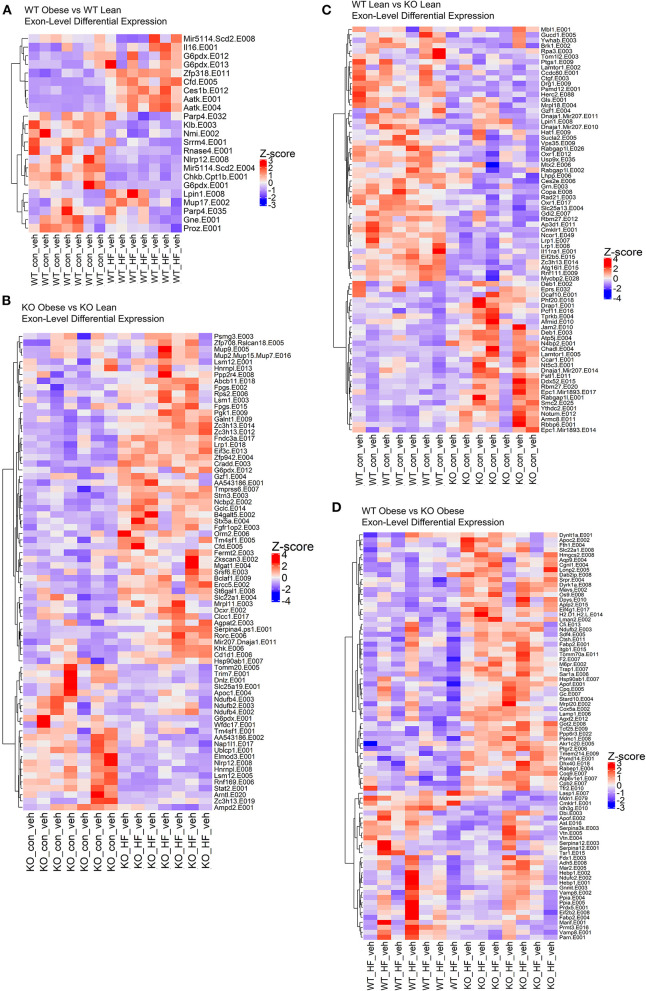
The loss of ChemR23 controls the transcriptional landscape at the exon level. **(A)** Heatmap of differentially expressed exons between WT obese and WT lean mice. **(B)** Heatmap of differentially expressed exons between ChemR23 KO obese and ChemR23 KO lean mice. **(C)** Heatmap of differentially expressed exons between WT lean and ChemR23 KO lean mice. **(D)** Heatmap of differentially expressed exons between WT obese and ChemR23 KO obese mice. All heatmaps are scaled by Z-score to center all the gene expression values on the same scale. Data are from 7 mice per group.

We conducted exon-level analyses between WT obese and lean mice. We found exon transcripts that mimicked the gene-level results with exons in genes such as Cfd, Aatk, and Scd; however, we see a more precise depiction of transcriptional regulation in the context of a high fat diet. For instance, in the gene-level analysis we found that Scd1 and Scd3 were significantly decreased in obese WT mice compared to lean WT mice. However, in the exon-level analysis we see a more complete picture where we also found that exon transcripts in the Scd2 gene encode miRNAs (miR-5114 on exon 8 and exon 4) that are also significantly decreased in the obese WT mice (Figure 3A). Therefore, we observe that multiple gene transcripts in the Scd gene family are differentially expressed. When comparing obese KO mice with lean KO mice, the obese KOs had upregulated differentially expressed exons largely contributing to pathways in NAFLD and oxidative phosphorylation, like in the gene-level analysis, but unlike the gene-level results PPAR signaling and complement/coagulation cascades were also upregulated in the exon-level results (Figure 3B). Significantly downregulated exons in the obese KO mice encompassed fatty acid degradation & metabolism pathways, proteosome and peroxisome complexes, and protein processing in the endoplasmic reticulum (Figure 3B).

Subsequently, we examined the effects of the ChemR23 KO on exon-level transcripts. We identified 202 downregulated exon transcripts and 72 upregulated exon transcripts in the lean KO mice compared to the lean wildtype mice (Figure 3C). Most of the downregulated exon transcripts in the knockout lean mice classified into pathways involving infections such as hepatitis B and influenza A, Jak-STAT signaling, and protein processing in the endoplasmic reticulum. To further determine effects of the ChemR23 KO in the context of a high fat diet, we examined differentially expressed exons that significantly differed between obese WT and obese KO mice. However, unlike the gene-level analysis where close to no differentially expressed genes were detected, in the exon-level analysis we found 82 differentially expressed exon transcripts that were largely upregulated in the obese KO mice compared to the obese WT mice (Figure 3D). Many of these upregulated exon transcripts comprised of lipid transport and binding, cholesterol metabolism, proteolysis, and fibrinolysis.

**Figure 4:**
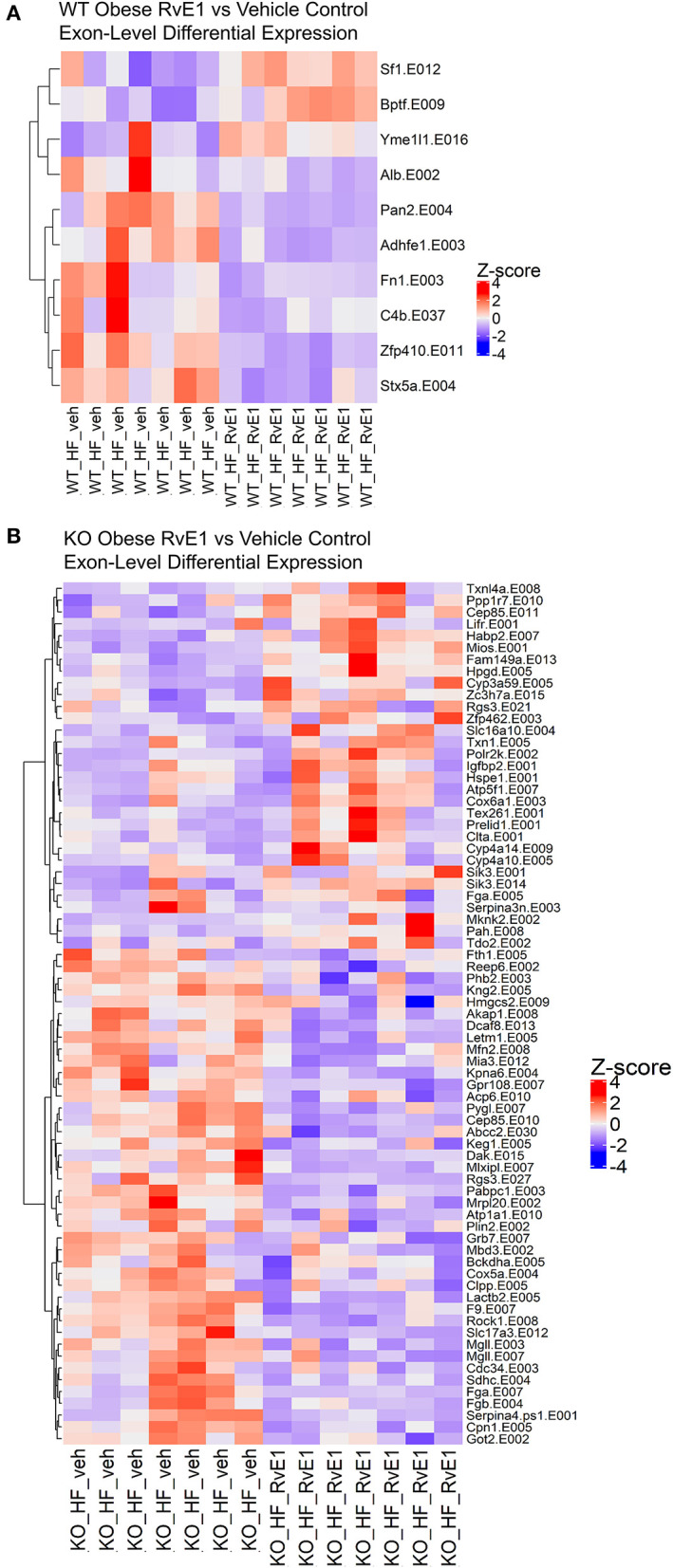
RvE1 controls transcriptional regulation and expression at the exon level. **(A)** Heatmap of differentially expressed exons between WT obese+RvE1 treated mice and WT obese+vehicle control treated mice. **(B)** Heatmap of differentially expressed exons between ChemR23 KO obese+RvE1 treated mice and ChemR23 KO obese+vehicle control treated mice. Data are from 7 mice per group.

In the obese WT RvE1 treated mice, compared to obese mice given a vehicle control, few exons were differentially expressed and were mostly downregulated, such as complement C4B (C4b, Log2FC = −0.88) and fibronectin 1 (Fn1, Log2FC = −0.81) (Figure 4A). In the obese KO RvE1 treated mice, relative to obese KO mice given vehicle control, there were 73 differentially expressed exons, 45 of which were downregulated and 28 upregulated with RvE1 treatment (Figure 4B). The downregulated exon transcripts mostly clustered into immunological pathways involving complement cascades, platelet activation, and fibrin clot formation. The upregulated exon transcripts clustered into the functions of metal ion and amino acid binding. Overall, these data highlight the importance of exon-level analyses to provide more in-depth characterization of dysregulated transcripts that may not be captured at the gene level. Furthermore, the findings again show some differential effects on exon transcripts upon the loss of ChemR23 and upon RvE1 administration between WT and ChemR23 KO obese mice.

### Exon Level Analyses Reveal Differential Expression of miRNAs Between Lean and Obese ChemR23 KO Mice

**Figure 5:**
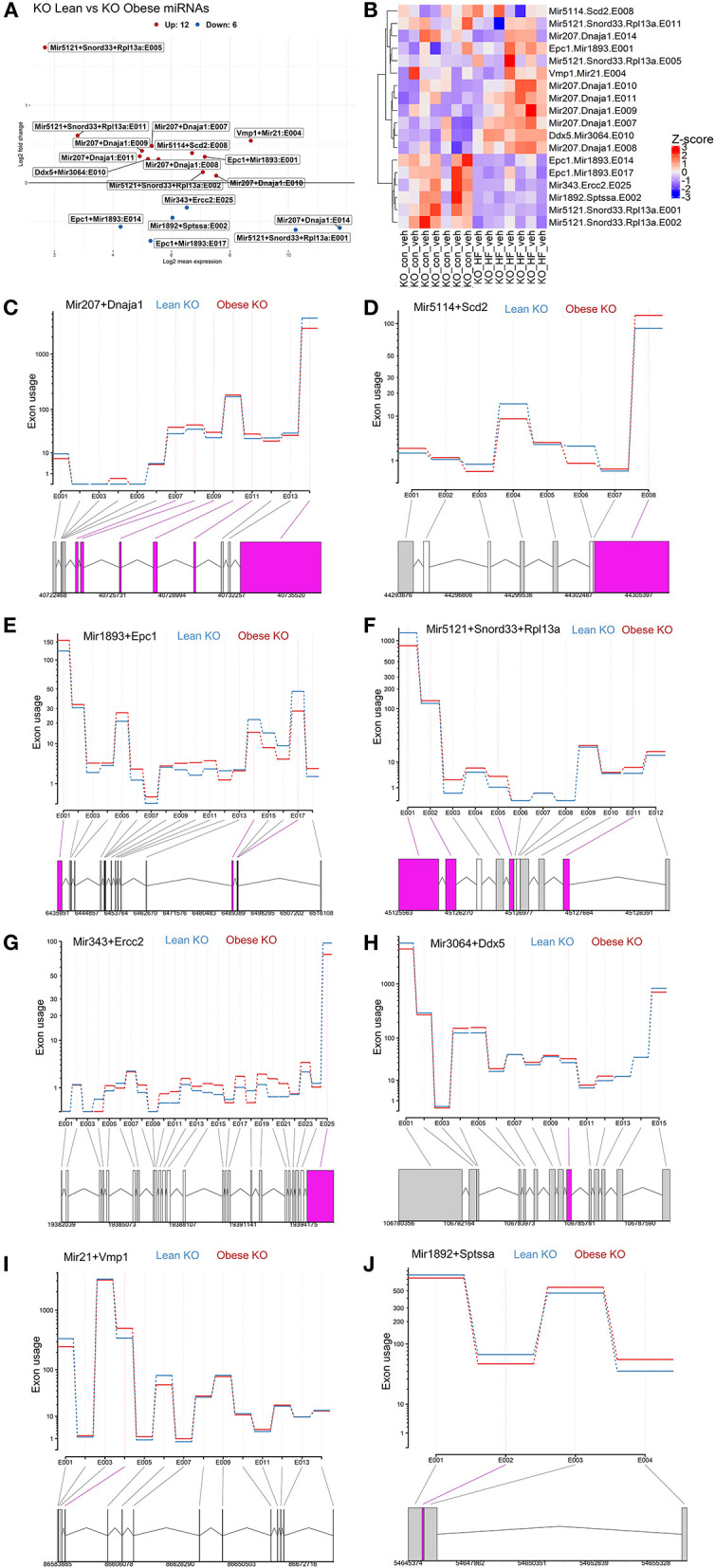
Exon level analyses show differential expression of mirnas between lean and obese ChemR23 KO mice. **(A)** MA plot between ChemR23 KO lean and ChemR23 KO obese mice showing miRNAs with a BH-adjusted p-value below 0.1 and Log2 fold change (FC) above one plotted by Log2FC in the y-axis and Log2 mean expression in the x-axis. Upregulated miRNAs are colored in red and downregulated miRNAs are colored in blue. **(B)** Heatmap of miRNAs with corresponding exons that were differentially expressed between ChemR23 KO lean and ChemR23 KO obese mice. Differential expression plot of exon transcripts in **(C)** miR-207 **(D)** miR-5114 **(E)** miR-1893, **(F)** miR-5121, **(G)** miR-343, **(H)** miR-3064, **(I)** miR-21, and **(J)** miR-1892 that were significantly different between ChemR23 KO lean and ChemR23 KO obese mice. Data are from 7 mice per group.

Next, we further conducted exon-level analyses between lean and obese ChemR23 KO mice. We discovered that comparing the lean ChemR23 KO mice with the obese ChemR23 KO mice yielded the most differentially expressed miRNAs than all the other groups. We found 12 significantly upregulated miRNA transcripts and 6 significantly downregulated miRNA transcripts in obese KO mice compared to lean KO mice (Figures 5A,B). Most the differentially expressed exon transcripts fell into miR-207 in the dnaJ heat shock protein family (Hsp40) member A1 (Dnaja1) gene, comprising of 6 differentially expressed exons with exon 14 containing the largest transcript segment, and all the miR-207 exons were upregulated in obese KO mice relative to lean KO mice (Figure 5C). For miR-5114 in the Scd2 gene there was one differentially expressed exon that was significantly decreased in the obese KO mice compared to the lean KO mice (Figure 5D). MiR-1893 in the enhancer of polycomb homolog 1 (Epc1) gene contained three differentially expressed exons, two of which (exons 14 and 17) were significantly downregulated in obese KO mice compared to lean KOs, and exon 1 was significantly upregulated in the obese KO mice (Figure 5E). For miR-5121 it spanned the small nucleolar RNA, C/D box 33 (Snord33) and ribosomal protein L13a (Rpl13a) genes with four differentially expressed exons, two of which were downregulated (exons 1–2) and two were upregulated (exons 5 and 11) in the obese KO mice versus the lean KOs (Figure 5F). MiR-343 contained in the excision repair cross-complementation group 2 (Ercc2) gene had only one differentially expressed transcript (exon 25) that was downregulated in the obese KO mice relative to lean KOs (Figure 5G). In miR-3064, contained in the DEAD-Box helicase 5 (Ddx5) gene, there was also one differentially expressed transcript (exon 10) that was upregulated in the obese KO mice compared to lean KOs (Figure 5H). MiR-21 spanned the vacuole membrane protein 1 (Vmp1) with one differentially expressed transcript (exon 4) that is mostly upregulated in the obese KO mice compared to lean KOs (Figure 5I). Lastly, miR-1892 contained in the serine palmitoyltransferase small subunit A (Sptssa) gene had one differentially expressed transcript at exon 2 that is downregulated in the obese KO mice relative to lean KO mice (Figure 5J). These results underscore the role of miRNAs in ChemR23 mediated signaling pathways, which may be relevant upon activation of ChemR23 with RvE1.

### Predicted Targets of miRNA Transcripts in KO Obese and KO Lean Mice

**Figure 6:**
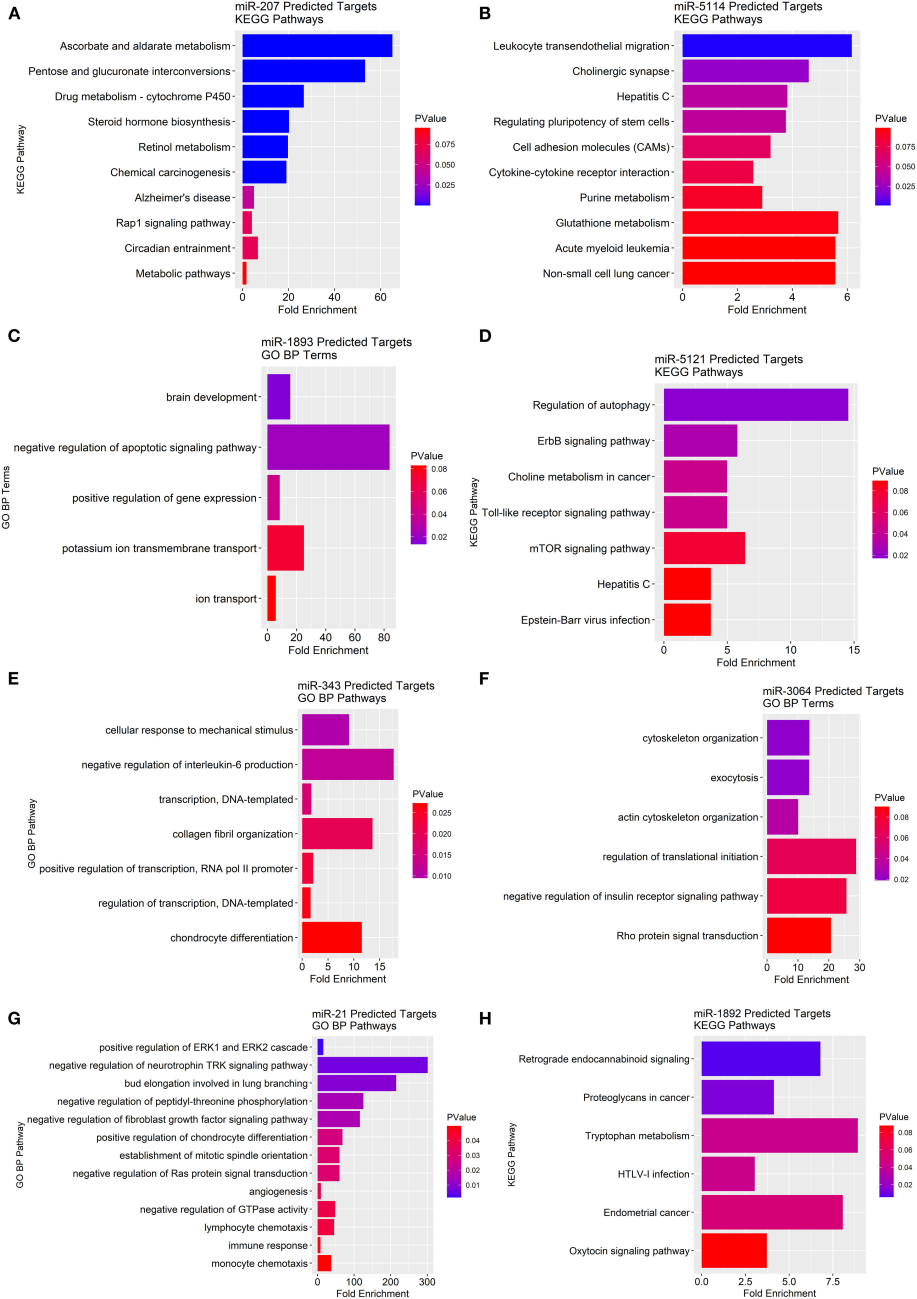
KEGG pathway and gene ontology analyses of predicted miRNA gene targets in ChemR23 KO obese and ChemR23 KO lean mice. Predicted miRNA KEGG pathway or gene ontology (GO) biological process (BP) targets are plotted by fold enrichment and colored by p-value, with red denoting the least significance and blue denoting the most significance. Predicted KEGG pathway targets for **(A)** miR-207 and **(B)** miR-5114 between ChemR23 KO lean and ChemR23 KO obese mice Predicted GO BP targets for **(C)** miR-1893 between ChemR23 KO lean and ChemR23 KO obese mice. Predicted KEGG pathway targets for **(D)** miR-5121 between ChemR23 KO lean and ChemR23 KO obese mice. Predicted GO BP targets for **(E)** miR-343, **(F)** miR-3064, and **(G)** miR-21 between ChemR23 KO lean and ChemR23 KO obese mice. Predicted KEGG pathway targets for **(H)** miR-1892 between ChemR23 KO lean and ChemR23 KO obese mice. Data are from 7 mice per group.

To determine the potential gene-pathway targets of the miRNAs that were identified, we conducted a miRNA target prediction analysis and clustered the generated gene lists into their corresponding KEGG pathways. For miRNAs with too few gene targets to cluster into KEGG pathways we report “biological process” gene ontologies instead. We found miR-207 likely targets genes in pathways mainly related to ascorbate/aldarate metabolism, cytochrome P450 metabolism, steroid hormone biosynthesis, and retinol metabolism (Figure 6A). For miR-5114 multiple immunological pathway targets were revealed such as leukocyte migration, cell adhesion molecules (CAMs), and cytokine-cytokine receptor interactions (Figure 6B). As for miR-1893, the primary gene ontology the gene targets classified into was apoptotic signaling with the largest fold enrichment at 83.7 followed by potassium ion transport at 25.1 (Figure 6C). For miR-5121 regulation of autophagy, ErbB signaling, choline metabolism, toll-like receptor signaling, and mTOR signaling were the primary pathway targets (Figure 6D). MiR-5121 also had a few targets in immunological pathways such as the hepatitis C and Epstein-Barr viral infection response. Similarly, miR-343 also had immunologically related gene targets such as negative regulation of IL-6 production (Figure 6E). On the other hand, miR-3064 primarily contained gene targets that classified into cytoskeletal functions such as exocytosis, cytoskeletal organization, and actin cytoskeletal filaments (Figure 6F). In addition, miR-3064 contained potential targets in negative regulation of the insulin receptor signaling pathway and Rho protein signal transduction. For miR-21, we found immunological pathway hits such as ERK1/ERK2 cascades, lymphocyte and monocyte chemotaxis, negative regulation of GTPases, and negative regulation of fibroblast growth factor signaling (Figure 6G). Lastly, miR-1892 had gene targets that spanned pathways such as retrograde endocannabinoid signaling, tryptophan metabolism, and cancer-related pathways (Figure 6H). Taken together, many predicted miRNA targets span a wide range of metabolic and inflammatory pathways that provide a framework for testing their role in ChemR23 mediated signaling.

## Discussion

This study provides the first in-depth transcriptional analysis of RvE1-ChemR23 mediated hepatic pathways that span gene and exon-level transcriptional changes. In the gene-level analyses, we first tested diet-level changes between groups and found that both WT and ChemR23 KO obese mice had dysregulated pathways related to insulin signaling, glucose intolerance, hepatic steatosis, and NAFLD compared to lean WT and ChemR23 KO controls. This indicates that the loss of ChemR23 is not protective against the transcriptional dysregulation that is driven by a high fat diet.

When comparing genotype driven differences between the WT lean and ChemR23 KO lean mice, we found that pathways related to activated immunological and chemotaxis markers were primarily upregulated in ChemR23 KO animals consuming a control diet. This is in agreement with multiple studies that have shown ChemR23's regulatory effects in immune cell chemotaxis and inflammation (13, 17, 57–60). Interestingly, when challenged with a high fat diet, the WT and KO obese mice portray the same gene-level profile, potentially indicating that obesity drives the two groups to have similar hepatic transcriptional landscapes when examining gene-level changes. However, with an exon-level analysis accounting for changes in alternative splicing, the gene-level analysis failed to detect differentially expressed exons in pathways such as lipid transport and cholesterol metabolism that are upregulated in the livers of obese ChemR23 KO mice. Therefore, these studies suggest that the loss of ChemR23 could be further exacerbating the hepatic transcriptome in response to a high fat diet. There is some evidence that the loss ChemR23 promotes a modest increase in body weight in older mice without a major impact on adipocyte differentiation (61). Thus, future studies should investigate the role of ChemR23 in liver lipid and glucose metabolism.

An interesting finding was that obese WT mice, relative to lean controls, had a robust reduction in pathways related to the biosynthesis of unsaturated fatty acids. We also observed a reduction in stearoyl-coA desaturase gene expression, consistent with the notion that unsaturated fatty acids are lowered in the livers of obese mice. This is worth exploring in greater detail in the future as a high fat diet may be rendering the liver to be deficient in key unsaturated fatty acids such as EPA and DHA and thereby downstream metabolites of these fatty acids including RvE1, which are critical players in resolution of inflammation (62). Indeed, there is evidence that liver Δ5- and Δ6-desaturase activities are lowered with a high fat diet, which has implications for the development of insulin resistance (63).

The gene-level analysis showed that WT obese RvE1 treated mice had significantly upregulated genes in pathways relating to insulin sensitivity, which corroborates our previous findings that established RvE1's effects at reducing hyperinsulinemia and hyperglycemia (10, 64). In the obese ChemR23 KO RvE1 treated mice, genes related to hepatic glucose homeostasis, such as Foxq1, were downregulated as well; however, decreasing Foxq1 is associated with increased impairment of glucose homeostasis (50). This is also worth further investigation as our previous findings showed that ChemR23 KO mice do not show improvements in hyperglycemia/insulinemia upon RvE1 treatment, indicating that RvE1's effects are ChemR23 dependent (10). Hence, in the obese KO mice, we found genes supporting mechanisms related to glucose impairment, indicating that in the absence of the ChemR23 receptor, RvE1's glucose homeostatic effects in the liver are impaired.

Exon level analyses of RvE1 treated mice revealed multiple exon transcripts related to immunological functions such as complement cascades. These pathways were downregulated in both obese WT and KO RvE1 treated mice, indicating that some of RvE1's immunological effects in the liver are also mediated by ChemR23-independent mechanisms. One likely mechanism would be RvE1 binding and signaling through the LTB_4_ receptor (BLT1), a major driver of inflammation (17, 65–67). Future studies will need to tease apart how RvE1 may control the inflammatory status at the cellular level by signaling through LTB_4_ across metabolic tissues such as liver and white adipose tissue. LTB_4_ is of interest given its role as a chemoattractant for pro-inflammatory cells (67). Overall, RvE1 is likely targeting multiple pathways related to hepatic chronic inflammation which could also include a role for chemerin, the ligand for ChemR23. Indeed, there is evidence for differing SPMs to control varying aspects of hepatic inflammation (68).

Another advancement from this study was the differential expression of miRNAs that were identified between lean ChemR23 KO vs. obese ChemR23 KO mice that would have not been detected with only a gene-level analysis. We identified miRNAs that target a variety of pathways. Many of these miRNA pathway targets related to immunological outcomes such as leukocyte and monocyte migration/chemotaxis, cell adhesion molecules, toll-like receptor signaling, cytokine receptor signaling, viral infection response, and negative regulation of IL-6 production. A limitation to the miRNA analysis is that we cannot determine whether the identified miRNA pathway targets are up- or downregulated since some miRNAs result in either inhibition or upregulation of transcription. However, these findings indicate that miRNAs can play a major role in ChemR23 mediated signaling, particularly in immunologically related phenotypes or inflammation, and should be considered in mechanistic studies related to the ChemR23 receptor and it's ligands (i.e., RvE1, chemerin), especially in the context of diet-induced obesity.

There are several limitations to this study. We relied on a four day treatment with RvE1, which does not model how RvE1 intake would occur in humans. Thus, future studies will need to examine how long-term RvE1 intake can control the transcriptional landscape in WT and KO mice. In addition, we did not examine sex differences as there is an increasing appreciation that SPM bioavailability is different between males and females (9, 69, 70). In addition, the effects of RvE1's parent compound EPA on glucose homeostasis also appear to be sex-specific (71). Furthermore, we did not conduct studies at the cellular level, which will provide insight on how the RvE1-ChemR23 axis controls the transcriptional landscape, particularly at the exon level of distinct cell types that express high levels of ChemR23. Future studies will need to connect if dietary EPA exerts its effects through RvE1 on the hepatic transcriptional landscape. This will require investigating the bioavailability of RvE1 in the liver in response to EPA administration. Finally, subsequent studies will need to probe the transcriptional response to RvE1 immediately after intervention with this SPM. In this study, we measured the transcriptional response a day after the last dose of RvE1. However, since RvE1 is rapidly metabolized, it is possible that transcriptional activity would vary at an earlier time point.

In conclusion, our study emphasizes the importance of measuring both whole gene level and in-depth exon level changes to capture a more accurate snapshot of transcriptional regulation that may not detected or lost in gene-level only analyses (72). Importantly, we discovered that the loss of ChemR23 is associated with a dysregulated transcriptional profile at the exon level in response to a high fat diet compared to controls. In addition, RvE1 administration upregulated genes related to pathways of insulin sensitivity in WT mice, which was generally not recapitulated with the ChemR23 KO mice given RvE1. These results provide inflammatory and metabolic targets of interest for future mechanistic studies focused on the RvE1-ChemR23 axis.

## Data Availability Statement

The datasets presented in this study can be found in online repositories. The names of the repository/repositories and accession number(s) can be found at: https://www.ncbi.nlm.nih.gov/, GSE188599.

## Ethics Statement

The animal study was reviewed and approved by University of North Carolina at Chapel Hill IACUC.

## Author Contributions

AA-S designed and performed research, analyzed data, and wrote and edited the paper. AP designed and performed research. SRS designed research, wrote parts of the paper, and edited the paper. All authors contributed to the article and approved the submitted version.

## Funding

This work was supported by NIH R01AT008375 (SRS) and P30DK05635 (SRS). This material was also based upon work supported by the National Science Foundation Graduate Research Fellowship Program under (Grant No. 1650116 to AA-S).

## Author Disclaimer

Any opinions, findings, and conclusions or recommendations expressed in this material are those of the author(s) and do not necessarily reflect the views of the National Science Foundation.

## Conflict of Interest

The authors declare that the research was conducted in the absence of any commercial or financial relationships that could be construed as a potential conflict of interest.

## Publisher's Note

All claims expressed in this article are solely those of the authors and do not necessarily represent those of their affiliated organizations, or those of the publisher, the editors and the reviewers. Any product that may be evaluated in this article, or claim that may be made by its manufacturer, is not guaranteed or endorsed by the publisher.

## References

[1] Ostermann AI West AL Schoenfeld K Browning LM Walker CG Jebb SA. Plasma oxylipins respond in a linear dose-response manner with increased intake of EPA and DHA: results from a randomized controlled trial in healthy humans. Am J Clin Nutr. (2019) 109:1251–63. 10.1093/ajcn/nqz016 31006007

[2] Al-Shaer AE Buddenbaum N Shaikh SR. Polyunsaturated fatty acids, specialized pro-resolving mediators, and targeting inflammation resolution in the age of precision nutrition. Biochim Biophys Acta Mol Cell Biol Lipids. (2021) 1866:158936. 10.1016/j.bbalip.2021.158936 33794384 PMC8496879

[3] Basil MC Levy BD. Specialized pro-resolving mediators: endogenous regulators of infection and inflammation. Nat Rev Immunol. (2016) 16:51–67. 10.1038/nri.2015.4 26688348 PMC5242505

[4] Chandrasekharan JA Sharma-Walia N. Lipoxins: nature's way to resolve inflammation. J Inflamm Res. (2015) 8:181–92. 10.2147/JIR.S90380 26457057 PMC4598198

[5] Serhan CN Sheppard KA. Lipoxin formation during human neutrophil-platelet interactions. Evidence for the transformation of leukotriene A4 by platelet 12-lipoxygenase *in vitro*. J Clin Invest. (1990) 85:772–80. 10.1172/JCI114503 2155925 PMC296494

[6] Serhan CN Chiang N Dalli J Levy BD. Lipid mediators in the resolution of inflammation. Cold Spring Harb Perspect Biol. (2014) 7:a016311. 10.1101/cshperspect.a016311 25359497 PMC4315926

[7] Kosaraju R Guesdon W Crouch MJ Teague HL Sullivan EM Karlsson EA. B cell activity is impaired in human and mouse obesity and is responsive to an essential fatty acid upon murine influenza infection. J Immunol. (2017) 198:4738–52. 10.4049/jimmunol.1601031 28500069 PMC5482422

[8] Neuhofer A Zeyda M Mascher D Itariu BK Murano I Leitner L. Impaired local production of proresolving lipid mediators in obesity and 17-HDHA as a potential treatment for obesity-associated inflammation. Diabetes. (2013) 62:1945–56. 10.2337/db12-0828 23349501 PMC3661630

[9] Crouch MJ Kosaraju R Guesdon W Armstrong M Reisdorph N Jain R. Frontline Science: a reduction in DHA-derived mediators in male obesity contributes toward defects in select B cell subsets and circulating antibody. J Leukoc Biol. (2019) 106:241–57. 10.1002/JLB.3HI1017-405RR 30576001 PMC10020993

[10] Pal A Al-Shaer AE Guesdon W Torres MJ Armstrong M Quinn K. Resolvin E1 derived from eicosapentaenoic acid prevents hyperinsulinemia and hyperglycemia in a host genetic manner. FASEB J. (2020) 34:10640–56. 10.1096/fj.202000830R 32579292 PMC7497168

[11] Tang Y Zhang MJ Hellmann J Kosuri M Bhatnagar A Spite M. Proresolution therapy for the treatment of delayed healing of diabetic wounds. Diabetes. (2013) 62:618–27. 10.2337/db12-0684 23043160 PMC3554373

[12] Laguna-Fernandez A Checa A Carracedo M Artiach G Petri MH Baumgartner R. ERV1/ChemR23 signaling protects against atherosclerosis by modifying oxidized low-density lipoprotein uptake and phagocytosis in macrophages. Circulation. (2018) 138:1693–705. 10.1161/CIRCULATIONAHA.117.032801 29739755 PMC6200387

[13] van der Vorst EPC Mandl M Müller M Neideck C Jansen Y Hristov M. Hematopoietic ChemR23 (Chemerin Receptor 23) fuels atherosclerosis by sustaining an m1 macrophage-phenotype and guidance of plasmacytoid dendritic cells to murine lesions-brief report. Arterioscler Thromb Vasc Biol. (2019) 39:685–93. 10.1161/ATVBAHA.119.312386 30786742

[14] Herrera BS Hasturk H Kantarci A Freire MO Nguyen O Kansal S. Impact of resolvin E1 on murine neutrophil phagocytosis in type 2 diabetes. Infect Immun. (2015) 83:792–801. 10.1128/IAI.02444-14 25486994 PMC4294250

[15] Sima C Montero E Nguyen D Freire M Norris P Serhan CN. ERV1 overexpression in myeloid cells protects against high fat diet induced obesity and glucose intolerance. Sci Rep. (2017) 7:12848. 10.1038/s41598-017-13185-7 28993702 PMC5634420

[16] Goralski KB McCarthy TC Hanniman EA Zabel BA Butcher EC Parlee SD. Chemerin, a novel adipokine that regulates adipogenesis and adipocyte metabolism. J Biol Chem. (2007) 282:28175–88. 10.1074/jbc.M700793200 17635925

[17] Arita M Ohira T Sun Y-P Elangovan S Chiang N Serhan CN. Resolvin E1 selectively interacts with leukotriene B4 receptor BLT1 and ChemR23 to regulate inflammation. J Immunol. (2007) 178:3912–7. 10.4049/jimmunol.178.6.3912 17339491

[18] Zhang J Wang M Ye J Liu J Xu Y Wang Z. The anti-inflammatory mediator resolvin e1 protects mice against lipopolysaccharide-induced heart injury. Front Pharmacol. (2020) 11:203. 10.3389/fphar.2020.00203 32256344 PMC7094758

[19] Largen PG French JE Moustaid-Moussa N Voruganti VS Mayer-Davis EJ Bizon CA. Synergizing mouse and human studies to understand the heterogeneity of obesity. Adv Nutr Res. (2021) 12:2023–34. 10.1093/advances/nmab040 33885739 PMC8483969

[20] Langmead B Salzberg SL. Fast gapped-read alignment with Bowtie 2. Nat Methods. (2012) 9:357–9. 10.1038/nmeth.1923 22388286 PMC3322381

[21] Liao Y Smyth GK Shi W. featureCounts: an efficient general purpose program for assigning sequence reads to genomic features. Bioinformatics. (2014) 30:923–30. 10.1093/bioinformatics/btt656 24227677

[22] Love MI Huber W Anders S. Moderated estimation of fold change and' ' dispersion for RNA-seq data with DESeq2. Genome Biol. (2014) 15:550. 10.1186/s13059-014-0550-8 25516281 PMC4302049

[23] Anders S Reyes A Huber W. Detecting differential usage of exons from RNA-seq data. Genome Res. (2012) 22:2008–17. 10.1101/gr.133744.111 22722343 PMC3460195

[24] Agarwal V Bell GW Nam J-W Bartel DP. Predicting effective microRNA target sites in mammalian mRNAs. Elife. (2015) 12:4. 10.7554/eLife.05005 26267216 PMC4532895

[25] Pan Y Lu L Chen J Zhong Y Dai Z. Identification of potential crucial genes and construction of microRNA-mRNA negative regulatory networks in osteosarcoma. Hereditas. (2018) 155:21. 10.1186/s41065-018-0061-9 29760609 PMC5941338

[26] Kirpich IA Gobejishvili LN Bon Homme M Waigel S Cave M Arteel G. Integrated hepatic transcriptome and proteome analysis of mice with high-fat diet-induced nonalcoholic fatty liver disease. J Nutr Biochem. (2011) 22:38–45. 10.1016/j.jnutbio.2009.11.009 20303728 PMC3860361

[27] Meng L-B Shan M-J Qiu Y Qi R Yu Z-M Guo P. TPM2 as a potential predictive biomarker for atherosclerosis. Aging. (2019) 11:6960–82. 10.18632/aging.102231 31487691 PMC6756910

[28] Heintz MM McRee R Kumar R Baldwin WS. Gender differences in diet-induced steatotic disease in Cyp2b-null mice. PLoS ONE. (2020) 15:e0229896. 10.1371/journal.pone.0229896 32155178 PMC7064244

[29] Song N-J Kim S Jang B-H Chang S-H Yun UJ Park K-M. Small molecule-induced complement factor D (adipsin) promotes lipid accumulation and adipocyte differentiation. PLoS ONE. (2016) 11:e0162228. 10.1371/journal.pone.0162228 27611793 PMC5017651

[30] Hoekstra M Stitzinger M van Wanrooij EJA Michon IN Kruijt JK Kamphorst J. Microarray analysis indicates an important role for FABP5 and putative novel FABPs on a Western-type diet. J Lipid Res. (2006) 47:2198–207. 10.1194/jlr.M600095-JLR200 16885566

[31] Fernández Gianotti T Burgueño A Gonzales Mansilla N Pirola CJ Sookoian S. Fatty liver is associated with transcriptional downregulation of stearoyl-CoA desaturase and impaired protein dimerization. PLoS ONE. (2013) 8:e76912. 10.1371/journal.pone.0076912 24098813 PMC3786952

[32] Yang H Herring J Wynn A Elison W Walton C Good L. Full body loss of the nuclear hormone receptor Nr4a3 results induces obesity and glucose intolerance. FASEB J. (2020) 34:1–1. 10.1096/fasebj.2020.34.s1.05965

[33] Zhang Y Li Y Niepel MW Kawano Y Han S Liu S. Targeted deletion of thioesterase superfamily member 1 promotes energy expenditure and protects against obesity and insulin resistance. Proc Natl Acad Sci USA. (2012) 109:5417–22. 10.1073/pnas.1116011109 22427358 PMC3325675

[34] Sun Y Yang Y Qin Z Cai J Guo X Tang Y. The acute-phase protein orosomucoid regulates food intake and energy homeostasis via leptin receptor signaling pathway. Diabetes. (2016) 65:1630–41. 10.2337/db15-1193 27207522

[35] Zhu Y Zhao S Deng Y Gordillo R Ghaben AL Shao M. Hepatic GALE regulates whole-body glucose homeostasis by modulating Tff3 expression. Diabetes. (2017) 66:2789–99. 10.2337/db17-0323 28877911 PMC5652600

[36] Dudek M Kołodziejski PA Pruszyńska-Oszmałek E Sassek M Ziarniak K Nowak KW. Effects of high-fat diet-induced obesity and diabetes on Kiss1 and GPR54 expression in the hypothalamic-pituitary-gonadal (HPG) axis and peripheral organs (fat, pancreas and liver) in male rats. Neuropeptides. (2016) 56:41–9. 10.1016/j.npep.2016.01.005 26853724

[37] El-Assaad W El-Kouhen K Mohammad AH Yang J Morita M Gamache I. Deletion of the gene encoding G0/G 1 switch protein 2 (G0s2) alleviates high-fat-diet-induced weight gain and insulin resistance, and promotes browning of white adipose tissue in mice. Diabetologia. (2015) 58:149–57. 10.1007/s00125-014-3429-z 25381555 PMC5001162

[38] Karlstetter M Walczak Y Weigelt K Ebert S Van den Brulle J Schwer H. The novel activated microglia/macrophage WAP domain protein, AMWAP, acts as a counter-regulator of proinflammatory response. J Immunol. (2010) 185:3379–90. 10.4049/jimmunol.0903300 20709948

[39] Tantawy MA Hatesuer B Wilk E Dengler L Kasnitz N Weiß S. The interferon-induced gene Ifi27l2a is active in lung macrophages and lymphocytes after influenza A infection but deletion of Ifi27l2a in mice does not increase susceptibility to infection. PLoS ONE. (2014) 9:e106392. 10.1371/journal.pone.0106392 25184786 PMC4153650

[40] Chen K Bao Z Tang P Gong W Yoshimura T Wang JM. Chemokines in homeostasis and diseases. Cell Mol Immunol. (2018) 15:324–34. 10.1038/cmi.2017.134 29375126 PMC6052829

[41] DePaula-Silva AB Gorbea C Doty DJ Libbey JE Sanchez JMS Hanak TJ. Differential transcriptional profiles identify microglial- and macrophage-specific gene markers expressed during virus-induced neuroinflammation. J Neuroinflammation. (2019) 16:152. 10.1186/s12974-019-1545-x 31325960 PMC6642742

[42] Yang YB Wu XL Ke B Huang YJ Chen SQ Su YQ. Effects of caloric restriction on peroxisome proliferator-activated receptors and positive transcription elongation factor b expression in obese rats. Eur Rev Med Pharmacol Sci. (2017) 21:4369–78. 29077158

[43] Orian A Eisenman RN. TGF-beta flips the Myc switch. Sci STKE. (2001) 2001:pe1. 10.1126/stke.2001.88.pe1 11752658

[44] Frederick JP Liberati NT Waddell DS Shi Y Wang X-F. Transforming growth factor beta-mediated transcriptional repression of c-myc is dependent on direct binding of Smad3 to a novel repressive Smad binding element. Mol Cell Biol. (2004) 24:2546–59. 10.1128/MCB.24.6.2546-2559.2004 14993291 PMC355825

[45] Haque R Nazir A SMAD. Transcription Factor, Sma-9, Attunes TGF-β Signaling Cascade Towards Modulating Amyloid Beta Aggregation and Associated Outcome in Transgenic C. elegans Mol Neurobiol. (2016) 53:109–19. 10.1007/s12035-014-8988-y 25407930

[46] Li MJ Wang P Liu X Lim EL Wang Z Yeager M. GWASdb: a database for human genetic variants identified by genome-wide association studies. Nucleic Acids Res. (2012) 40:D1047–54. 10.1093/nar/gkr1182 22139925 PMC3245026

[47] Greenawalt DM Sieberts SK Cornelis MC Girman CJ Zhong H Yang X. Integrating genetic association, genetics of gene expression, and single nucleotide polymorphism set analysis to identify susceptibility Loci for type 2 diabetes mellitus. Am J Epidemiol. (2012) 176:423–430. 10.1093/aje/kws123 22865700 PMC3499116

[48] Athinarayanan S Fan Y-Y Wang X Callaway E Cai D Chalasani N. Fatty Acid Desaturase 1 Influences Hepatic Lipid Homeostasis by Modulating the PPARα-FGF21 Axis. Hepatol Commun. (2021) 5:461–77. 10.1002/hep4.1629 33681679 PMC7917273

[49] Jimenez V Jambrina C Casana E Sacristan V Muñoz S Darriba S. FGF21 gene therapy as treatment for obesity and insulin resistance. EMBO Mol Med. (2018) 10:e8791. 10.15252/emmm.201708791 29987000 PMC6079533

[50] Cui Y Qiao A Jiao T Zhang H Xue Y Zou Y. The hepatic FOXQ1 transcription factor regulates glucose metabolism in mice. Diabetologia. (2016) 59:2229–39. 10.1007/s00125-016-4043-z 27421728

[51] Nunemaker CS Chung HG Verrilli GM Corbin KL Upadhye A Sharma PR. Increased serum CXCL1 and CXCL5 are linked to obesity, hyperglycemia, and impaired islet function. J Endocrinol. (2014) 222:267–76. 10.1530/JOE-14-0126 24928936 PMC4135511

[52] Palmer DC Guittard GC Franco Z Crompton JG Eil RL Patel SJ. Cish actively silences TCR signaling in CD8+ T cells to maintain tumor tolerance. J Exp Med. (2015) 212:2095–113. 10.1084/jem.20150304 26527801 PMC4647263

[53] Delconte RB Kolesnik TB Dagley LF Rautela J Shi W Putz EM. CIS is a potent checkpoint in NK cell-mediated tumor immunity. Nat Immunol. (2016) 17:816–24. 10.1038/ni.3470 27213690

[54] Sawant KV Poluri KM Dutta AK Sepuru KM Troshkina A Garofalo RP. Chemokine CXCL1 mediated neutrophil recruitment: Role of glycosaminoglycan interactions. Sci Rep. (2016) 6:33123. 10.1038/srep33123 27625115 PMC5021969

[55] Boles NC Hirsch SE Le S Corneo B Najm F Minotti AP. NPTX1 regulates neural lineage specification from human pluripotent stem cells. Cell Rep. (2014) 6:724–36. 10.1016/j.celrep.2014.01.026 24529709

[56] Deng C Xiang Y Tan T Ren Z Cao C Liu B. The imbalance of B-lymphocyte subsets in subjects with different glucose tolerance: relationship with metabolic parameter and disease status. J Diabetes Res. (2017) 2017:5052812. 10.1155/2017/5052812 28491871 PMC5410374

[57] Herová M Schmid M Gemperle C Hersberger M. ChemR23, the receptor for chemerin and resolvin E1, is expressed and functional on M1 but not on M2 macrophages. J Immunol. (2015) 194:2330–7. 10.4049/jimmunol.1402166 25637017

[58] López-Vicario C Rius B Alcaraz-Quiles J González-Périz A Martínez-Puchol AI Casulleras M. Association of a variant in the gene encoding for ERV1/ChemR23 with reduced inflammation in visceral adipose tissue from morbidly obese individuals. Sci Rep. (2017) 7:15724. 10.1038/s41598-017-15951-z 29146976 PMC5691181

[59] Wittamer V Franssen J-D Vulcano M Mirjolet J-F Le Poul E Migeotte I. Specific recruitment of antigen-presenting cells by chemerin, a novel processed ligand from human inflammatory fluids. J Exp Med. (2003) 198:977–85. 10.1084/jem.20030382 14530373 PMC2194212

[60] Iannone F Lapadula G. Chemerin/ChemR23 pathway: a system beyond chemokines. Arthritis Res Ther. (2011) 3:104. 10.1186/ar3273 21542878 PMC3132019

[61] Rouger L Denis GR Luangsay S Parmentier M. ChemR23 knockout mice display mild obesity but no deficit in adipocyte differentiation. J Endocrinol. (2013) 219:279–89. 10.1530/JOE-13-0106 24084834

[62] González-Périz A Horrillo R Ferré N Gronert K Dong B Morán-Salvador E. Obesity-induced insulin resistance and hepatic steatosis are alleviated by omega-3 fatty acids: a role for resolvins and protectins. FASEB J. (2009) 23:1946–57. 10.1096/fj.08-125674 19211925 PMC2698663

[63] Valenzuela R Barrera C Espinosa A Llanos P Orellana P Videla LA. Reduction in the desaturation capacity of the liver in mice subjected to high fat diet: Relation to LCPUFA depletion in liver and extrahepatic tissues. Prostaglandins Leukot Essent Fatty Acids. (2015) 98:7–14. 10.1016/j.plefa.2015.04.002 25910408

[64] Sima C Paster B Van Dyke TE. Function of pro-resolving lipid mediator resolvin E1 in type 2 diabetes. Crit Rev Immunol. (2018) 38:343–65. 10.1615/CritRevImmunol.2018026750 30806214 PMC6392080

[65] Saeki K Yokomizo T. Identification, signaling, and functions of LTB4 receptors. Semin Immunol. (2017) 33:30–6. 10.1016/j.smim.2017.07.010 29042026

[66] Subramanian BC Majumdar R Parent CA. The role of the LTB4-BLT1 axis in chemotactic gradient sensing and directed leukocyte migration. Semin Immunol. (2017) 33:16–29. 10.1016/j.smim.2017.07.002 29042024 PMC8288505

[67] Li P Oh DY Bandyopadhyay G Lagakos WS Talukdar S Osborn O. LTB4 promotes insulin resistance in obese mice by acting on macrophages, hepatocytes and myocytes. Nat Med. (2015) 21:239–47. 10.1038/nm.3800 25706874 PMC4429798

[68] Clària J Flores-Costa R Duran-Güell M López-Vicario C. Proresolving lipid mediators and liver disease. Biochim Biophys Acta Mol Cell Biol Lipids. (2021) 1866:159023. 10.1016/j.bbalip.2021.159023 34352389

[69] Rathod KS Kapil V Velmurugan S Khambata RS Siddique U Khan S. Accelerated resolution of inflammation underlies sex differences in inflammatory responses in humans. J Clin Invest. (2017) 127:169–82. 10.1172/JCI89429 27893465 PMC5199722

[70] English JT Norris PC Hodges RR Dartt DA Serhan CN. Identification and profiling of specialized pro-resolving mediators in human tears by lipid mediator metabolomics. Prostaglandins Leukot Essent Fatty Acids. (2017) 117:17–27. 10.1016/j.plefa.2017.01.004 28237084 PMC5329889

[71] Pal A Sun S Armstrong M Manke J Reisdorph N Adams VR. Beneficial effects of eicosapentaenoic acid on the metabolic profile of obese female mice entails upregulation of HEPEs and increased abundance of enteric Akkermansia muciniphila. Biochim Biophys Acta Mol Cell Biol Lipids. (2022) 1867:159059. 10.1016/j.bbalip.2021.159059 34619367 PMC8627244

[72] Al-Shaer AE Flentke GR Berres ME Garic A Smith SM. Exon level machine learning analyses elucidate novel candidate miRNA targets in an avian model of fetal alcohol spectrum disorder. PLoS Comput Biol. (2019) 15:e1006937. 10.1371/journal.pcbi.1006937 30973878 PMC6478348

