# Supplementary material for: Resolvin E1-ChemR23 Axis Regulates the Hepatic Metabolic and Inflammatory Transcriptional Landscape in Obesity at the Whole Genome and Exon Level
Source: Front Nutr. 2021 Dec 24;8:799492. doi: 10.3389/fnut.2021.799492 (PMC8740313; doi:10.3389/fnut.2021.799492)
Supplement: Supplementary file 9 [file Data_Sheet_9.DOCX]

https://www.ncbi.nlm.nih.gov/geo/query/acc.cgi?acc=GSE188599
